# New records of the soldier flies of Morocco with a bibliographical inventory of the North African fauna (Diptera, Stratiomyidae)

**DOI:** 10.3897/zookeys.709.13364

**Published:** 2017-10-18

**Authors:** Driss Yimlahi, Turgay Üstüner, Sanae Zinebi, Boutaïna Belqat

**Affiliations:** 1 Department of Biology, Faculty of Sciences, University Abdelmalek Essaâdi, Tétouan, 93030, Morocco; 2 Selçuk University, Faculty of Science, Department of Biology, Campus Alaeddin Keykubat, Selçuklu, 42075 Konya, Turkey

**Keywords:** Algeria, Checklist, Egypt, Morocco, North Africa, Rif, Soldier Flies, Stratiomyidae, Tunisia

## Abstract

A checklist of soldier flies species recorded from the North African countries of Morocco, Algeria, Tunisia, Libya, and Egypt is based on both literature records and material newly collected in Morocco. Four subfamilies (Stratiomyinae, Sarginae, Nemotelinae, and Pachygasterinae), and twelve species from five genera have been collected and are recognized in Morocco. *Pachygaster atra* (Panzer, 1798), *Oxycera pardalina* (Meigen, 1822), *Nemotelus danielssoni* (Mason, 1989), and *Oxycera terminata* (Meigen, 1822) are newly recorded to the North African fauna. *Nemotelus atriceps* (Loew, 1856) and *Nemotelus maculiventris* (Bigot, 1861) are reported for the first time in Morocco. The present number of soldier flies known from Morocco is 33.

## Introduction

Stratiomyids (soldier flies) constitute one of the moderately large families of Diptera that exhibit an extreme array of morphological diversity, as well as a moderate range of life histories, with about 400 genera and about 2,700 species currently recognised worldwide (http://www.catalogueoflife.org/col/browse/tree/id/2bda68991ddbf2b55b5c7e66c8a125ad). The soldier flies are found all over the world, but are particularly diverse in tropical regions (Woodley 2001, 2011).

The family comprises varied members, ranging from 2.0 to 20.0 mm in length. While some species are entirely slender, others are stout or evidently flattened, with coloration ranging from strikingly patterned dark with a yellow, sometimes white or greenish pattern and frequent metallic reflections (Rozkošný 1997), to rather dull and concolourous dark brownish black (Figs 22, 23).

Important studies have been done over the world, like those of Rozkošný (1977, 1982, 1983, 1998, 2004) who specially studied the European Stratiomyidae, Rozkošný and Nartshuk (1988) who provided a catalogue of the soldier flies and Woodley (2001, 2011) who provided the world catalogue of the soldier flies.

To date, the soldier fly fauna of Morocco has been poorly investigated. The first records of Moroccan Stratiomyidae were given by Becker and Stein (1912) in their study of the Diptera of Morocco. For the next two decades, they received sporadic study devoted mainly, to the records of Moroccan species amongst the Diptera of Morocco or among the Stratiomyidae of the Palaearctic Region (Séguy 1930; Lindner 1936). Till now, no comprehensive and specific study has been published on Moroccan stratiomyids.

In this present study, 12 species of soldier flies have been recorded from 23 sampling sites in Morocco (Table 1), including a total of 83 specimens (47 males and 36 females). We report the first occurrences of the species *Nemotelus atriceps* (Loew, 1856) and *Nemotelus maculiventris* (Bigot, 1861) in Morocco, in addition to *Pachygaster atra* (Panzer, 1798), *Oxycera pardalina* (Meigen, 1822), *Oxycera terminata* (Meigen, 1822) and *Nemotelus danielssoni* (Mason, 1989) for the first time not only in Morocco but for the North African continent.

**Table 1.** Sampling sites (in alphabetical order) harbouring the species collected in Morocco with localities, geographical coordinates and altitudes.

Site	Locality	Province	Altitude (m)	Geographical coordinates
**Rif Mountains**				
1. Affluent Tarmast	Parc National d’Al Hoceima	Al Hoceima	168	35°10.666'N, 004°03.088'W
2. Aïn El Malâab	Parc National Talassemtane	Chefchaouen	1278	35°05.509'N, 005°09.443'W
3. Aïn Sidi Brahim Ben Arrif	Route Moulay Abdessalam	Larache	897	35°20.398'N, 005°32.712'W
4. Barrage Moulay Bouchta	Larbaa Beni Hassan	Tétouan	364	35°15.864'N, 005°21.221'W
5. Cascade Chrafate	Chrafate	Chefchaouen	820	35°03.997'N, 005°06.434'W
6. Daya Afrate	Tanaqoub	Chefchaouen	600	35°05.634'N, 005°26.028'W
7. Daya Aïn Jdioui	Aïn Jdioui	Tanger-Assilah	5	35°34.074'N, 005°55.499'W
8. Daya Mezine	Mezine	Chefchaouen	778	35°06.104'N, 005°21.177'W
9. Daya Rmali	El Malâab, Parc National Talassemtane	Chefchaouen	1276	35°05.563'N, 005°09.488'W
10. Daya Tazia	Route Moulay Abdessalam	Larache	721	35°20.814'N, 005°33.139'W
11. Douar Kitane	Kitane	Tétouan	52	35°32.412'N, 005°20.393'W
12. Lac Ametrasse	Chrafate	Chefchaouen	828	35°05.014'N, 005°5.130'W
13. Oued Abou Bnar	Douar Abou Bnar, Parc National Talassemtane	Chefchaouen	1254	35°10.977'N, 005°08.005'W
14. Oued Achekrade	Douar Aouzighen	Tétouan	642	35°22.931'N, 005°20.364'W
15. Oued El Koub	Souk El Had	Chefchaouen	124	35°01.298'N, 005°25.333'W
16. Oued Izelfane	Beni Boufrah	Al Hoceima	206	35°07.272'N, 004°12.555'W
17. Oued Majjou (Hafa meqlouba)	Majjou	Chefchaouen	825	35°06.175'N, 005°10.836'W
18. Oued Zandoula	15 km au Nord de Ouazzane	Ouazzane	108	34°55.707'N, 005°31.942'W
19. Ruisseau Maison forestière	Parc National Talassemtane	Chefchaouen	1674	35°08.076'N, 005°08.262'W
20. Taghbalout	Larbaa Beni Hassan	Tétouan	379	35°15.323'N, 005°20.887'W
**Middle Atlas Mountains**				
21. Cascade Aïn Vittel	Aïn Vittel	Ifrane	1555	33°33.682'N, 005°07.463'W
22. Mchacha Aïn Vittel	Aïn Vittel	Ifrane	1585	33°33.206'N, 005°06.722'W
**Anti Atlas Mountains**				
23. Village Massa	Village Massa	Massa	24	29°59.353'N, 009’38.708'W

## Materials and methods

Three techniques were used to collect Stratiomyidae: rearing larvae and pupae in the laboratory from collected substrates in the field following the technique used by Afzan and Belqat (2016), and collecting adults with both sweep net and malaise traps. Samples were collected by two authors (DY and BB) and the specimens were either micro-pinned or preserved in ethyl alcohol. Because specimens of the subfamily Nemotelinae are small, usually less than 5 mm in length, study and illustration of the male and/or female terminalia required use of a microscope. Observation of detailed structures of the male and/or female terminalia was sometimes needed to confirm species identification. Dissections of terminalia were prepared using the methods and equipment described by (Nagatomi and Iwata 1976), then transferred to 70% alcohol where the internal tissues were removed with fine forceps. After washing, the dissected genital parts were preserved in (1:10) a mixture of glycerine and ethyl alcohol for observation.

**Figures 1–4.**
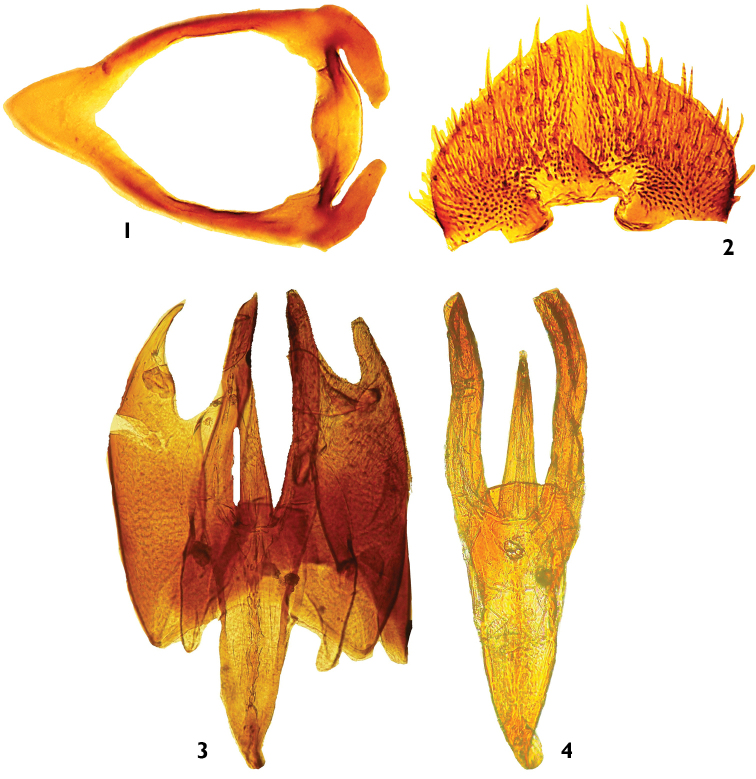
*Nemotelus danielssoni* female and male terminalia: **1** Genital furca **2** Subgenital plate **3** Genital capsule with Aedeagal complex (dorsal view) **4** Genital capsule with Median process (lateral view).

**Map 1.**
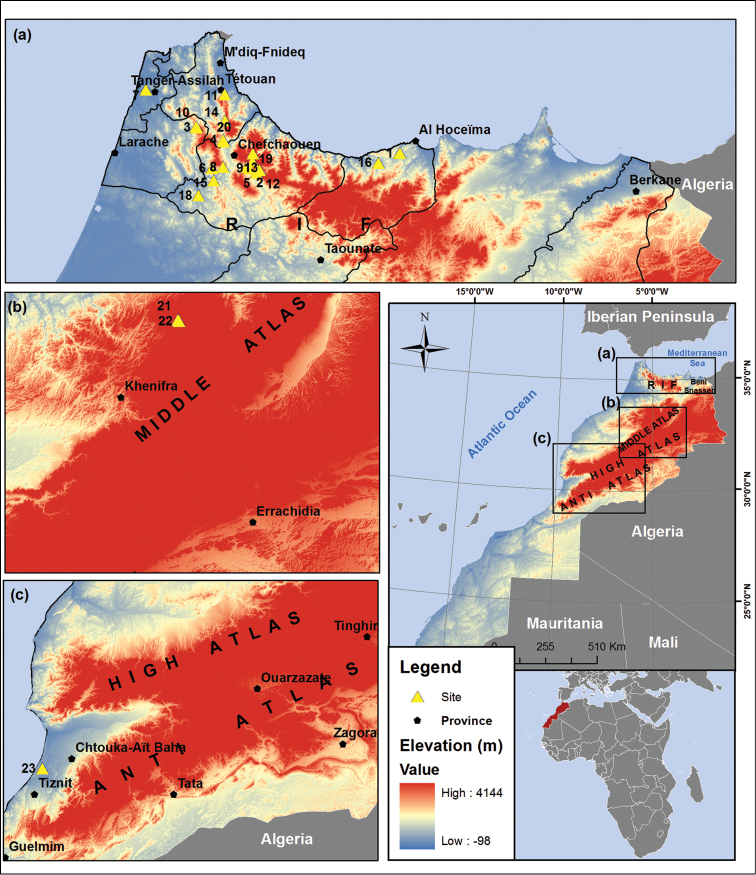
Map showing all sampling sites for soldier flies collected in this study in Morocco.

Preparations of the male and/or female terminalia, as well as the illustrations are given here (Figs 1–4). Species were recognised according to the identification keys of Séguy (1930, 1953), Rozkošný (1982, 1983) and Rozkošný (1977). A list of 23 sampling sites, with coordinates and altitudes, is presented in Table 1, and the locations of the sites are shown in Map 1, done using the logiciel GisArc (Geographic Information System, version 9.3). Photographs of the sampling localities showing Moroccan habitats of the species newly recorded are given (by DY and BB). All the material is deposited in the insect collection of the department of Biology, Faculty of Sciences, University Abdelmalek Essaâdi, Tétouan, Morocco.

The nomenclature and the list of the species known to North Africa follow the world Catalogue of the Stratiomyidae (Woodley 2001).

Table 2 summarises the species presently known from North Africa.

**Table 2.** The following checklist summarises the species of solider fly presently known from North Africa. Taxa are listed according to the classification scheme of Woodley (2001). Those species which are new records for North Africa are noted with (^) symbol, those new for Morocco are marked with (+), and the species which represent the new site in Morocco are with one asterisk (*).

Specimens	Morocco	Algeria	Tunisia	Libya	Egypt
Subfamily Beridinae					
*Beris rozkosnyi* Kassebeer, 1996	X				
*Chorisops tunisiae* (Becker, 1915)	X	X	X		
Subfamily Pachygastrinae					
*Aspidacantha atra* Kertesz, 1916					X
*Pachygaster atra* Panzer, 1798	X^				
*Pachygaster maura* Lindner, 1939	X				
Subfamily Clitellariinae					
*Adoxomyia flauipes* (Fabricius, 1798)		X			
*Pycnomalla aterrima* Sack, 1912	X	X			
*Pycnomalla auriflua* (Erichson, 1841)	X	X			
*Pycnomalla splendens* (Fabricius, 1787)	X	X			
Subfamily Sarginae					
*Chloromyia formosa* (Scopoli, 1763)	X*	X	X		
*Sargus bipunctatus* (Scopoli, 1763)			X		
Subfamily Stratiomyinae					
*Dicorymbimyia annulatus* (Becker, 1906)			X		
*Oxycera germanica* (Szilady, 1932)		X			
*Oxycera morrisii* (Curtis, 1833)		X			
*Oxycera ochracea* (Vaillant, 1950)		X			
*Oxycera orientalis* (Lindner, 1974)					X
*Oxycera pardalina* (Meigen, 1822)	X^				
*Oxycera rara* (Scopoli, 1763)		X			
*Oxycera tenebricosa* (Vaillant, 1952)		X			
*Oxycera torrentium* (Vaillant, 1950)		X			
*Oxycera terminata* Meigen, 1822	X^				
*Oxycera trilineata* (Linnaeus, 1767)	X*	X			
*Peritta melichlora* Becker, 1906		X			
*Vanoyia tenuicornis* Macquart, 1834	X				
*Odontomyia disciclara* (Séguy, 1929)		X			
*Odontomyia alolena* (Séguy, 1930)	X				
*Odontomyia angulata* (Panzer, 1798)	X	X			X
*Odontomyia discolor* (Loew, 1846)	X	X			
*Odontomyia flavissima* (Rossi 1790)	X	X	X		
*Odontomyia limbata* (Wiedemann, 1822)	X*	X	X		
*Odontomyia megacephala* Olivier, 1812					X
*Odontomyia microcera* (Séguy, 1930)	X				
*Odontomyia xanthopus* Bezzi, 1906					X
*Oplodontha minuta* Fabricius, 1794					X
*Oplodontha viridula* (Fabricius, 1775)		X			
*Stratiomyia africana* Szilady, 1941		X			
*Stratiomys cenisia* Meigen, 1822	X	X	X		X
*Stratiomys deserticolor* Lindner, 1930					X
*Stratiomys hispanica* Pleske, 1901		X			
*Stratiomys longicornis* (Scopoli, 1763)	X	X	X		X
*Stratiomys singularior* (Harris, 1776)					X
Subfamily Nemotelinae					
*Lasiopa benoisti* Séguy, 1930	X	X			
*Lasiopa manni* Mik, 1882			X		
*Lasiopa pantherina* Séguy, 1930	X				
*Nemotelus anchora* Loew, 1846		X	X		X
*Nemotelus atriceps* Loew, 1856	X+	X	X		
*Nemotelus beckeri* Hauser, 1998		X	X		
*Nemotelus candidus* Becker, 1906		X			X
*Nemotelus carthaginis* Becker, 1906			X		
*Nemotelus cingulatus* Dufour, 1852	X*	X	X		
*Nemotelus danielssoni* Mason, 1989	X^				
*Nemotelus dentatus* Becker, 1902					X
*Nemotelus lasiops* Loew, 1846			X		
*Nemotelus latiusculus* Loew, 1871	X*	X	X		
*Nemotelus longirostris* Wiedemann, 1824	X	X	X		
*Nemotelus maculiventris* Bigot, 1861	X+	X			
*Nemotelus marinus* Becker, 1902					X
*Nemotelus matrouhensis* Mohammad, Fadl, Gadalla & Badrawy, 2009					X
*Nemotelus nigrifrons* Loew, 1846	X*	X	X	X	
*Nemotelus niloticus* Olivier, 1811					X
*Nemotelus notatus* Zetterstedt, 1842					X
*Nemotelus pantherinus* (Linnaeus, 1758)	X				
*Nemotelus proboscideus* Loew, 1846		X	X	X	
*Nemotelus punctiventris* Becker, 1902					X
*Nemotelus subuliginosus* Rozkosny, 1974	X				
*Nemotelus ventralis* Meigen, 1830	X				
*Nemotelus nigrinus* Fallen, 1817	X				

## Results

### Faunistic records

#### Subfamily BERIDINAE

##### Genus *BERIS* Latreille, 1802

***Beris rozkosnyi* Kassebeer, 1996**

**North African literature records.** Morocco: Middle Atlas, Meknès, Ifrane (Kasebber 1996: 155; Woodley 2001: 66).

**World distribution**. Spain (Woodley 2001: 66).

##### Genus *CHORISOPS* Rondani, 1856

***Chorisops tunisiae* (Becker, 1915)**

= *Beris tunisiae* Becker, 1915

**North African literature records.** Morocco, Algeria, Tunisia: La Calle (Woodley 2001: 68).

**World distribution.** Portugal, Spain (Woodley 2001: 68); Sardinia (Mason et al. 2009: 509-510).

#### Subfamily PACHYGASTRINAE Loew, 1856

##### Genus *ASPIDACANTHA* Kertesz, 1916

***Aspidacantha atra* Kertesz, 1916**

**North African literature record.** Egypt (Lindner 1936: 211; Woodley 2001: 86).

**World distribution.** Palaearctic: Israel, Turkmenistan. Afrotropical: Ethiopia, Tanzania, Uganda, Zaire, Zimbabwe (Woodley 2001: 86); United Arab Emirates (Hauser 2008: 591–592).

##### Genus *PACHYGASTER* Meigen, 1803

***Pachygaster atra* Panzer, 1798**

= *Nemotelus ater* Panzer, 179

= *Sargvs pachygaster* Fallen, 1817

**Figure 5.**
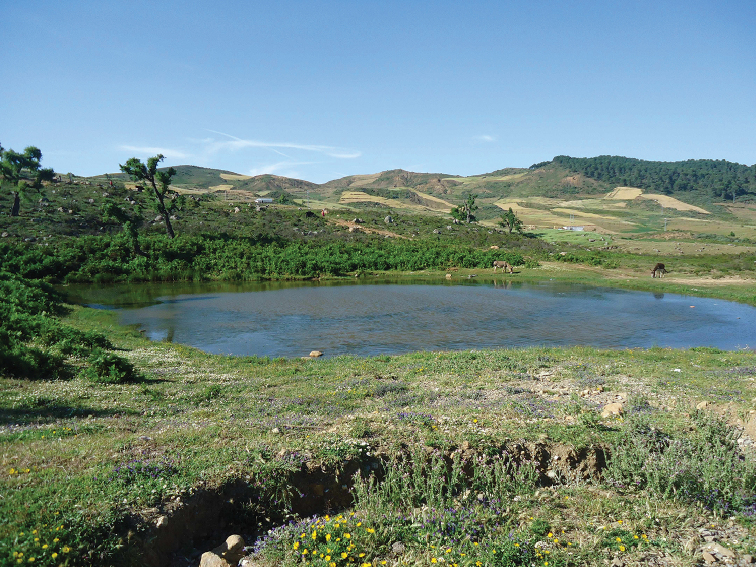
Habitat of *Pachygaster atra*: Daya Mezine.

**New record.** Morocco, Rif: Daya Mezine (Fig. 5), 1♀, 11/VI/2013, sweep net, Coll. Yimlahi and Belqat.

**World distribution.** Austria, Belgium, Bulgaria, Czech Republic, England, France, Georgia, Germany, Greece, Hungary, Ireland, Israel, Italy, Netherlands, Poland, Portugal, Romania, Russia, Scotland, Slovakia, Spain, Sweden, Switzerland, Turkey, Ukraine, Wales, Yugoslavia (Woodley 2001: 125).

***Pachygaster maura* Lindner, 1939**

**North African literature record.** Morocco (Lindner 1939: 314).

**World distribution.** Portugal, Spain (Woodley 2001: 126).

#### Subfamily CLITELLARIINAE

##### Genus *ADOXOMYIA* Kertesz, 1907

***Adoxomyia flauipes* (Fabricius, 1798)**

= *Stratiomys flauipes* Fabricius, 1798

= *Euparyphus kabylinus* Bigot, 1879b

**World distribution.** Only known in North African from Algeria: Oran, Tebessa (Lindner 1936: 158–159); Oran (Woodley 2001: 151).

##### Genus *PYCNOMALLA* Gerstaecker, 1857

***Pycnomalla aterrima* Sack, 1912**

**World distribution.** This species is known in North Africa only from Morocco: Middle Atlas and Algeria: Tizi s’Tkrine (Séguy 1930: 59); Morocco: Daiet Ahoua (Séguy 1953: 78); Morocco and Algeria (Woodley 2001: 172).

***Pycnomalla auriflua* (Erichson, 1841)**

= *Stratiomys auriflua* Erichson, 1841

**World distribution.** Known in North Africa only from Morocco: Middle Atlas and Algeria: Soufouloud (Séguy 1930: 59); Morocco, Algeria (Woodley 2001: 172).

***Pycnomalla splendens* (Fabricius, 1787)**

= *Stratiomys splendens* Fabricius, 1787

= *Ephippium rufitarse* Macquart, 1838

= Pycnomalla splendens ssp. jordanica Lindner, 1974

**North African literature records.** Morocco (Séguy 1930: 59, 1953: 78); Morocco, Tunisia (Lindner 1936: 149–150); Morocco, Algeria, Tunisia (Woodley 2001: 172).

**World distribution.** Armenia, Israel, Portugal, Spain (Woodley 2001: 172); Turkey (Üstüner et al. 2002: 23).

##### Subfamily SARGINAE

###### Genus *CHLOROMYIA* Duncan, 1837

***Chloromyia formosa* (Scopoli, 1763)**

= *Musca formosa* Scopoli, 1763

= *Nemotelus flavogeniculatus* De Geer, 1776

= *Musca cicur* M. Harris, 1778

= *Musca aurata* Fabricius, 1787

= *Sargus aeneus* Walckenaer, 1802

= *Sargus xanthopterus* Meigen, 1804

= *Sargus azureus* Loew, 1840

**Figure 6.**
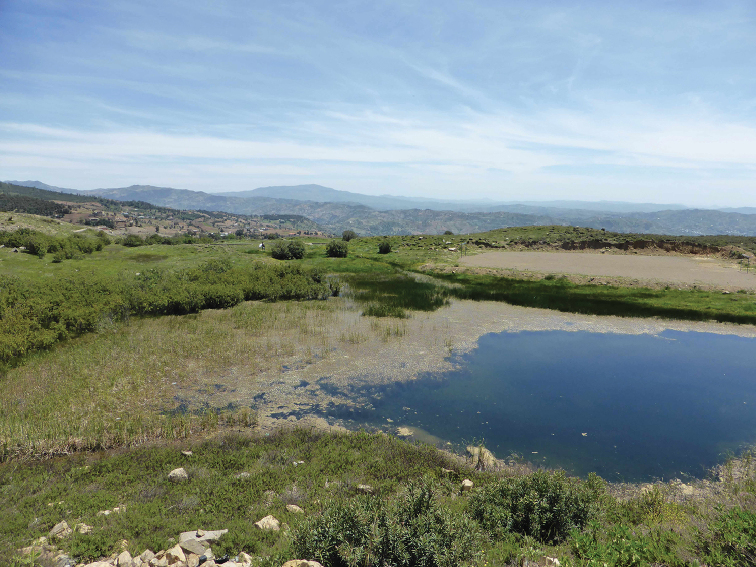
Habitat of *Chloromyia formosa*: Lac Ametrasse.

**New localities.** Morocco, Rif: Taghbalout, 1♂2♀♀, 5/IV/2014, sweep net, Coll. Yimlahi and Belqat; Lac Ametrasse (Fig. 6), 1♂, 24/V/2013, Coll. Yimlahi and Belqat, sweep net; Douar Kitane, 1♂, 10/IV/2014, Malaise trap, 1♀, 2/V/2014, sweep net, Coll. Yimlahi and Belqat.

**North African literature records.** Morocco: Tangier (Becker and Stein 1912: 64), Algeria: M’Rassine (Séguy 1930: 66), Merja Zerga (Pârvu et al. 2006); Morocco, Algeria, Tunisia (Woodley 2001: 189).

**World distribution.** Nearctic: USA (New York, introduced). Palaearctic, Austria, Bulgaria, Czech Republic, England, France, Germany, Greece, Italy, Poland, Portugal, Romania, Russia, Slovakia, Slovenija, Spain, Sweden, Switzerland, Turkey, Yugoslavia (Woodley 2001: 189); Sardinia (Mason et al. 2009: 508–509).

###### Genus *SARGUS* Fabricius, 1798

***Sargus bipunctatus* (Scopoli, 1763)**

= *Musca bipunctata* Scopoli, 1763

= *Sargus reaumuri* Meigen, 1804

= *Sargus reaumurii* Fabricius, 1805

= *Sargus sulphureus* Meigen, 1822

= *Sargus bipunctatus* O. Costa, 1844

= *Chrysochroma fasciatus* Szilady, 1929

= *Geosargus perpulcher* James, 1936

**North African literature record.** Tunisia (Woodley 2001: 221).

**World distribution.** Nearctic: Canada (British Columbia), USA (Oregon, Washington). Palaearctic: Albania, Austria, Belgium, Bulgaria, Croatia, Czech Republic, England, France, Georgia, Germany, Greece, Hungary, Ireland, Italy, Netherlands, Poland, Romania, Serbia, Slovakia, Slovenija, Switzerland (Woodley 2001: 221); Sardinia (Mason et al. 2009: 524).

##### Subfamily STRATIOMYINAE Latreille, 1802

###### Tribe OXYCERINI Enderlein, 1914

####### Genus *DICORYMBIMYIA* Woodley 2001

***Dicorymbimyia annulatus* (Becker, 1906)**

= *Oxycera annulata* Becker, 1906

**World distribution.** Tunisia: Zaghouan, Tunis (Woodley 2001: 240).

####### Genus *OXYCERA* Meigen, 1803

= *Hermione* Meigen, 1800

***Oxycera germanica* (Szilday , 1932)**

= *Hermione germanica* Szilady, 1932

= *Hermione dorieri* Vaillant, 1950

= Hermione dorieri var. barbarica Vaillant, 1950

**North African literature record.** Algeria: Aurès Mountains, Arris (Woodley 2001: 247).

**World distribution.** France, Germany, Switzerland (Woodley 2001: 247).

***Oxycera morrisii* (Curtis, 1833)**

= *Oxycera ranzonii* Schiner, 1857

= *Hermione muscaria ronzonii* Vaillant, 1950

= Hermione morrisi var. auresi Vaillant, 1950

= Hermione morrisi var. minuta Vaillant, 1950

**North African literature records.** Algeria: vicinity of Alger (Woodley 2001: 249).

**World distribution.** Austria, Belgium, Denmark, England, France, Germany, Greece, Ireland, Israel, Italy, Spain, Switzerland, Yugoslavia (Woodley 2001: 249).

***Oxycera ochracea* (Vaillant, 1950)**

= *Hermione ochracea* Vaillant, 1950

**World distribution.** Only known in North Africa from Algeria: Aurès Mountains, Arris, Constantine (Woodley 2001: 249).

***Oxycera orientalis* (Lindner, 1974)**

= *Heraclina orientalis* Lindner, 1974

= *Heraclina stigmosa orientalis* (Lindner & Freidberg, 1978)

**North African literature record.** Egypt (Badrawy 2006: 252).

**World distribution.** Israel (Woodley 2001: 249).

***Oxycera pardalina* (Meigen, 1822)**

= *Oxycera amoena* Loew, 1857

= *Oxycera engadinica* Jaennicke, 1866

= *Oxycera calceata* Loew, 1871

= *Hermione sahunica* Séguy, 1934

= Hermione pardalina var. oldenbergi Lindner, 1938

= Hermione pardalina var. nigrifrons Szilady, 1941

= Hermione morrisi var. bohemica Hrbacek, 1945

= *Hermione armata* Vaillant, 1950

= Hermione pardalina var. depressa Vaillant & Delhom, 1956

= Hermione pardalina var. alticola Vaillant & Delhom, 1956

**Figure 7.**
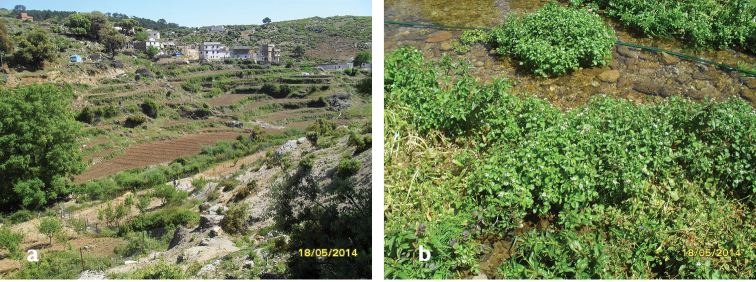
**a** Habitat of *Oxycera pardalina*: Oued Abou Bnar (from where the substrate is taken for rearing adults) **b** Habitat of *Oxycera pardalina*: Oued Abou Bnar environment.

**Figure 8.**
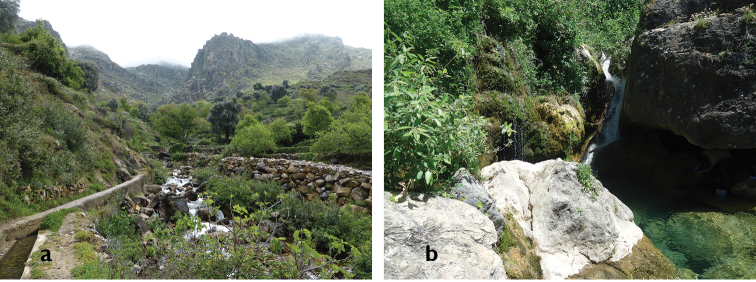
**a** Habitat of *Oxycera pardalina*: Oued Majjou (Hafa meqlouba) environment **b** Habitat of *Oxycera pardalina*: Oued Majjou (Hafa meqlouba).

**Figure 9.**
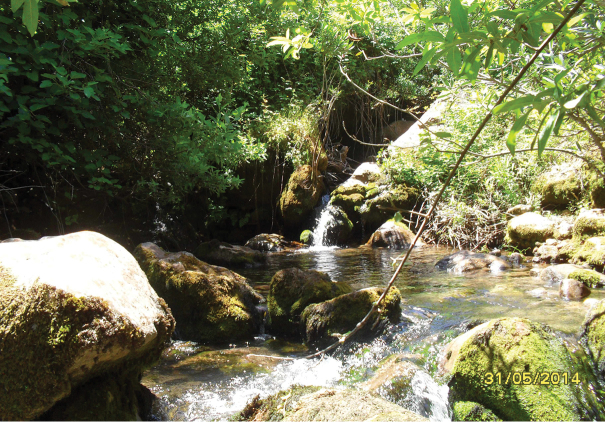
Habitat of *Oxycera pardalina*: Oued Achekrade.

**Figure 10.**
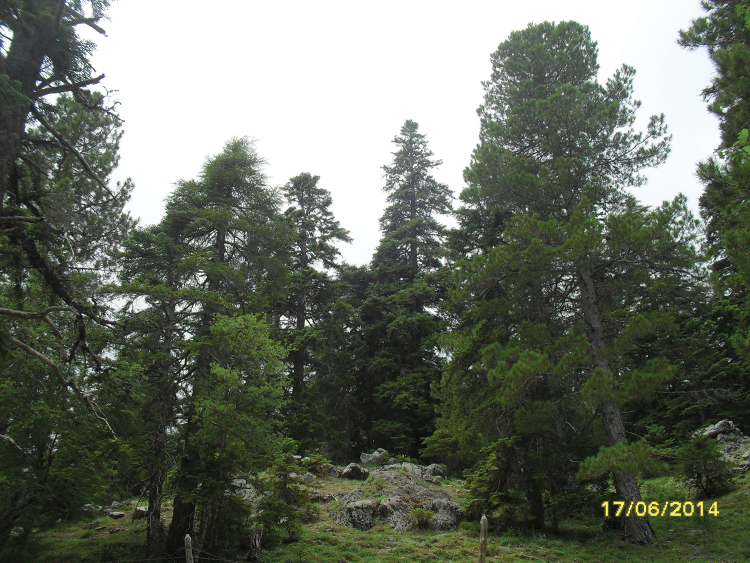
Habitat of *Oxycera pardalina*: Ruisseau Maison forestière environment.

**Figure 11.**
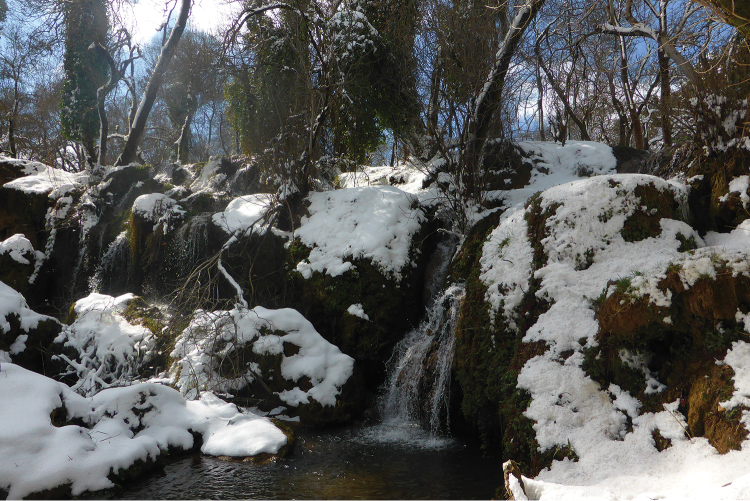
Habitat of *Oxycera pardalina*: Cascade AïnVittel (photograph taken on 17/II/2016, when the material was collected).

**Figure 12.**
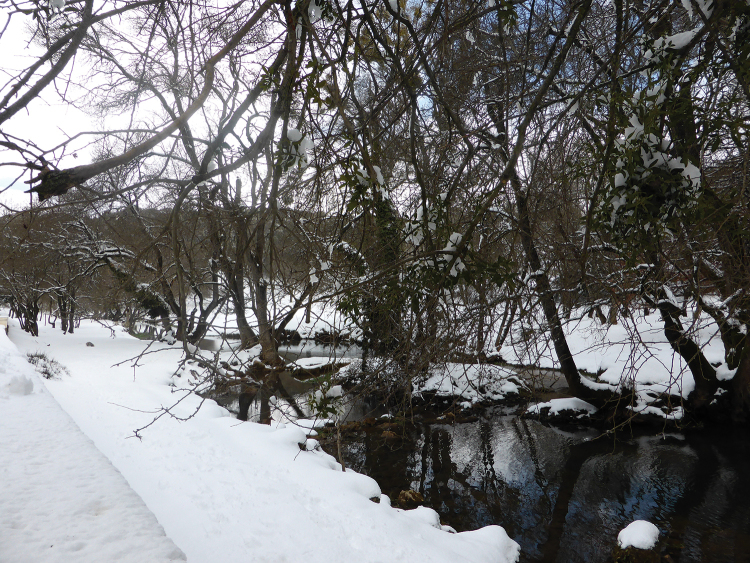
Habitat of *Oxycera pardalina*: Mchacha Aïn Vittel (photograph taken on 17/II/2016, when the material was collected).

**New records.** Oued Abou Bnar (Fig. 7a, b), 17/V/2014, sweep net, 1♀, 18/V/2014-12/VI/2014, reared, 2♂♂1♀; Oued Majjou (Hafa meqlouba) (Fig. 8a, b), 1♀, 9/IV/2015-15/VI/2015, reared; Oued Achekrade (Fig. 9), 1♀, 31/V/2014-8/VI/2014, reared; Ruisseau Maison forestière (Fig. 10), 2♀♀, 24/IV/2015, sweep net; Cascade Aïn Vittel (Fig. 11), 2♂♂1♀, 17/II/2016-20/IV/2016, reared; Mchacha Aïn Vittel (Fig. 12), 1♂, 17/II/2016-20/IV/2016, reared, Coll. Yimlahi and Belqat.

**World distribution.** Albania, Armenia, Austria, Belgium, Czech Republic, England, France, Georgia, Germany, Hungary, Ireland, Italy, Netherlands, Poland, Romania, Russia, Slovakia, Spain, Sweden, Switzerland, Yugoslavia (Woodley 2001: 250).

***Oxycera rara* (Scopoli, 1763)**

= *Musca rara* Scopoli, 1763

= *Musca tardigradus* M. Harris, 1778

= *Stratiomys maculata* Geoffroy *in* Fourcroy, 1785

= *Oxycera pulchella* Meigen, 1822

= Hermione pulchella var. similis (Vaillant, 1950)

**North African literature record.** Algeria: Aurès Mountains, Arris (Woodley 2001: 251)

**World distribution.** Andorra, Austria, Belgium, Czech Republic, England, France, Germany, Hungary, Italy, Netherlands, Poland, Romania, Slovakia, Slovenija, Spain, Switzerland, Wales, Yugoslavia (Woodley 2001: 251); Sardinia (Mason et al. 2009: 524).

***Oxycera tenebricosa* (Vaillant, 1952)**

= *Hermione tenebricosa* Vaillant, 1952

**World distribution.** Only known in North Africa from Algeria: Atlas de Blida (Woodley 2001: 252).

***Oxycera terminata* Meigen, 1822**

**Figure 13.**
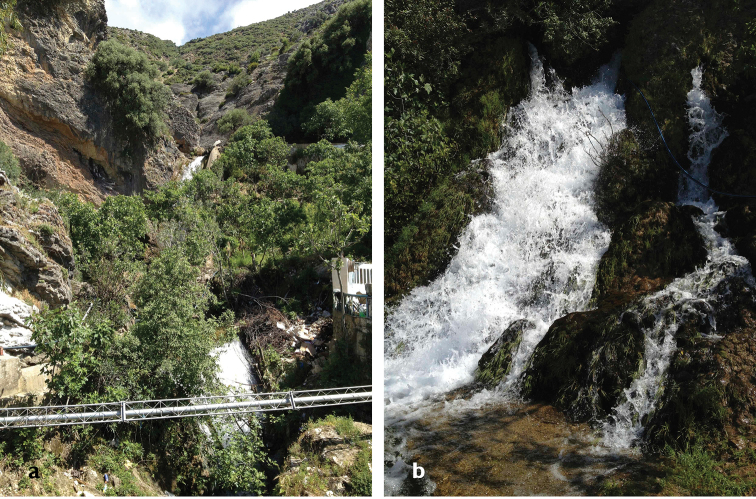
**a** Habitat of *Oxycera terminata*: Cascade Chrafate environment **b** Habitat of *Oxycera terminata*: Cascade Chrafate (extraction site of the substrate for rearing adults).

**New records.** Morocco, Rif: Cascade Chrafate (Fig. 13a, b), 2♂♂2♀♀, 18/III/2015-9/V/2015, 1♂, 18/III/2015-20/IV/2015, reared, Coll. Yimlahi and Belqat.

**World distribution.** Austria, Czech Republic, Denmark, England, France, Greece, Hungary, Poland, Romania, Slovakia, Switzerland, Yugoslavia (Woodley 2001: 252).

***Oxycera torrentium* (Vaillant, 1950)**

= *Hermione torrentium* Vaillant, 1950

**World distribution.** Only known in North Africa from Algeria: Atlas of Bilda, La Chiffa, Aurès Mountains (Woodley 2001: 252).

***Oxycera trilineata* (Linnaeus, 1767)**

= *Musca graeca* Pontoppidan, 1763

= *Musca trilineata* Linnaeus, 1767

= *Musca hypoleon* Linnaeus, 1767

= *Stratiomys fasciata* Geoffroy *in* Fourcroy, 1785

= *Oxycera proxima* Loew, 1873

= Oxycera trilineata var. collaris Brunetti, 1889

= Hermione trilineata ssp. transfasciata Pleske, 1925

= Hermione trilineata ssp. ferghanensis Pleske, 1925

= *Hermione ucrainica* Paramonov, 1926

= *Hermione bucheti* Séguy, 1930

= Hermione trilineata ssp. angustistomata Lindner, 1938

= Hermione trilineata var. biroi Szilady, 1941

= Hermione trilineata var. sajoi Szilady, 1941

= Hermione trilineata var. algira Vaillant, 1950

**Figure 14.**
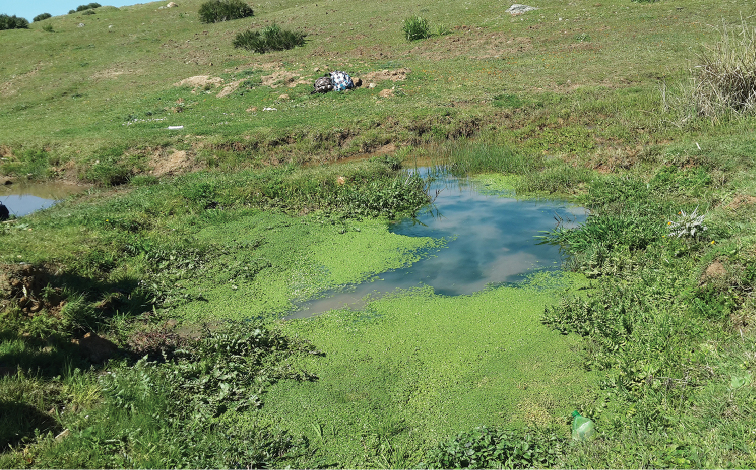
Habitat of *Oxycera trilineata* : Daya Aïn Jdioui.

**New locality.** Morocco, Rif: Daya Aïn Jdioui (Fig. 14), 1♂, 28/III/2015-27/V/2015, reared, Coll. Yimlahi and Belqat.

**North African literature records.** Morocco: Tangier (Becker and Stein 1912: 63, Séguy 1930: 62); Tangier, Algeria: Aurès Mountains, Arris (Woodley 2001: 252).

**World distribution.** Austria, Belgium, Bulgaria, China, Czech Republic, Denmark, England, Finland, France, Georgia, Germany, Greece, Hungary, Ireland, Israel, Italy, Kyrgyzstan, Latvia, Lithuania, Mongolia, Netherlands, Norway, Poland, Portugal, Romania, Russia, Scotland, Slovakia, Spain, Sweden, Switzerland, Turkey, Ukraine, Uzbekistan, Wales, Yugoslavia (Woodley 2001: 252); Sardinia (Mason et al. 2009: 524); United Arab Emirates (Hauser 2014: 695-697).

####### Genus *PERITTA* Becker, 1906

***Peritta melichlora* Becker, 1906**

**World distribution.** Only known in North Africa from Algeria: Biskra, spring at Hammam-Salahin (Woodley 2001: 254).

####### Genus *VANOYIA* Villeneuve, 1908

***Vanoyia tenuicornis* Macquart, 1834**

= *Oxycera nigra* Macquart, 1834

= *Oxycera longicornis* Dale, 1848

= *Oxycera longicornis* Walker, 1851

= *Vanoyia scutellata* Villeneuve, 1908

= *Vanoyea separata* Kertesz, 1921

**North African literature record.** Morocco: Tangier (Lindner 1936: 194, Woodley 2001: 254).

**World distribution.** Belgium, England, France, Ireland, Spain (Woodley 2001: 254).

###### Tribe STRATIOMYINI Latreille, 1802

####### Genus *ODONTOMYIA* Meigen, 1803

***Odontomyia alolena* (Séguy, 1930)**

= *Eulalia alolena* Séguy, 1930

**World distribution.** Only known in North Africa from Morocco: Casablanca, Tangier, Mahaidja, Aïn Leuh (Séguy 1930: 65, Woodley 2001: 269).

***Odontomyia angulata* (Panzer, 1798)**

= *Eulalia angulata* Panzer, 1798

= *Stratiomys angulata* Panzer, 1798

= *Stratiomys vulpina* Panzer, 1798

= *Stratiomys hydropota* Meigen, 1822

= *Odontomyia latifaciata* Macquart, 1834

= *Stratiomys brevicornis* Loew, 1840

= *Stratiomys brevicornis* Loew, 1840

= *Stratiomys ruficornis* Zetterstedt, 1842

= *Odontomyia hydrophila* Loew, 1846

**North African literature records.** Morocco: Tangier (Becker and Stein 1912: 62); Morocco, Algeria, Egypt (Woodley 2001: 269); Egypt (Badrawy 2006: 253).

**World distribution.** Afghanistan, Albania, Austria, Belgium, Bulgaria, China, Czech Republic, Denmark, England, Estonia, Finland, France, Germany, Greece, Hungary, Iran, Israel, Italy, Kazakhstan, Netherlands, Poland, Romania, Russia, Slovakia, Spain, Sweden, Switzerland, Turkey, Yugoslavia (Woodley 2001: 269); Sardinia (Mason et al. 2009: 521–522).

***Odontomyia disciclara* (Séguy, 1929)**

= *Eulalia disciclara* Séguy, 1929

**World distribution.** Only known in North African from Algeria: Touggourt (Lindner 1936: 87; Woodley 2001: 273).

***Odontomyia discolor* (Loew, 1846a)**

= Eulalia (Odontomyia) discolor Loew, 1897

= *Odontomyia limbata* Macquart *in* Lucas, 1849

**North African literature records.** Morocco: Tangier (Becker and Stein 1912: 62); Morocco, Algeria: Constantine (Woodley 2001: 274).

**World distribution.** Afghanistan, France, Greece, Israel, Italy, Kazakhstan, Kirgizia, Romania, Russia, Spain, Tajikistan, Turkey, Turkmenistan (Woodley 2001: 274); Sardinia (Mason et al. 2009: 522).

***Odontomyia flavissima* (Rossi 1790)**

= *Stratiomys flavissima* Rossi, 1790

= *Stratiomys decora* Wiedemann *in* Meigen, 1822

= *Stratiomys infoscata* Meigen, 1830

= *Odontomyia semiviolacea* Brullé, 1833

= *Odontomyia nigripes* Macquart, 1847

= *Odontomyia limbipennis* Macquart, 1847

= *Odontomyia laufferi* Strobl *in* Czemy & Strobl, 1909

**North African literature records.** Morocco, Algeria, Tunisia (Woodley 2001: 275).

**World distribution.** Albania, Austria, Azerbaijan, Bulgaria, Czech Republic, France, Georgia, Greece, Hungary, Israel, Italy, Kazakhstan, Portugal, Romania, Russia, Slovakia, Spain, Switzerland, Syria, Turkey, Yugoslavia (Woodley 2001: 275).

***Odontomyia limbata* (Wiedemann, 1822)**

= *Stratiomys limbata* Wiedemann *in* Meigen, 1822

= *Clitellaria pacifica* Wiedemann *in* Meigen, 1822

= *Opseogymnus flavo signata* A. Costa, 1857

**Figure 15.**
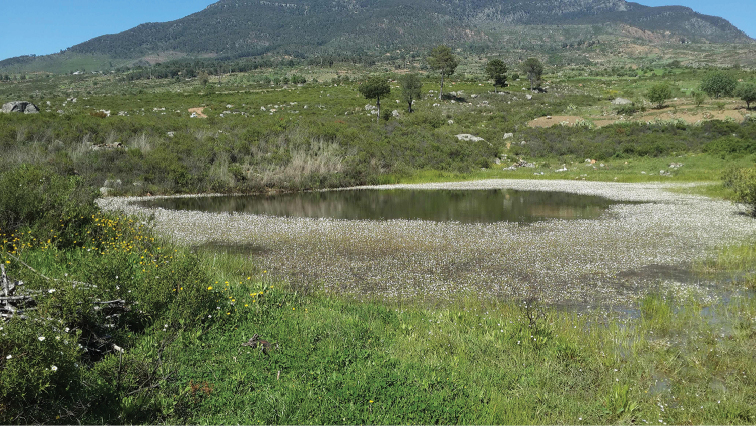
Habitat of *Odontomyia limbata* and *Nemotelus cingulatus*: Daya Afrate.

**Figure 16.**
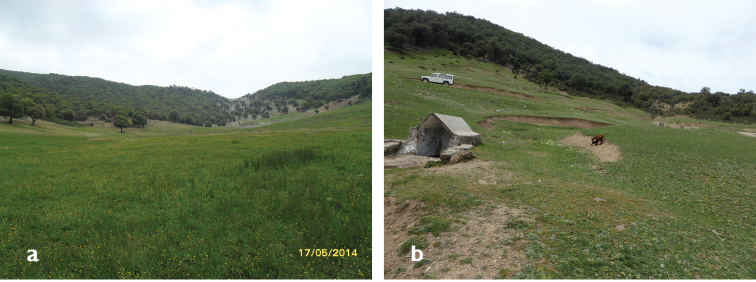
**a** Habitat of *Odontomyia limbata*: Aïn El Malâab **b** Habitat of *Odontomyia limbata*: Aïn El Malâab environment.

**New locality.** Morocco, Rif: Lac Ametrasse, 2♂♂1♀, 28/IV/2015; Aïn Sidi Brahim Ben Arrif, 4♂♂, 23/IV/2015; Daya Afrate (Fig. 15), 1♀, 18/IV/2015; Ruisseau Maison forestière, 11♂1♀, 8/V/2014; Aïn El Malâab (Fig. 16a, b), 1♂2♀♀, 17/V/2014; Daya Rmali El Malâab, 1♀, 17/V/2014, Daya Tazia, 2♂♂1♀, 12/V/2015, sweep net, Coll. Yimlahi and Belqat.

**North African literature records.** Morocco: Tangier (Becker and Stein 1912: 62); Middle Atlas, Tangier, Algeria (Séguy 1930: 65); Morocco, Algeria, Tunisia (Woodley 2001: 278).

**World distribution.** France, Italy, Portugal, Spain (Woodley 2001: 278).

***Odontomyia megacephala* Olivier, 1812**

**World distribution.** Only known in North Africa from Egypt (Lindner 1936: 92; Badrawy 2006: 253), Nil river (Woodley 2001: 279).

***Odontomyia microcera* (Séguy, 1930)**

= *Eulalia microcera* Séguy, 1930

**World distribution.** Only known in North Africa from Morocco: Meknès (Séguy 1930: 65; Woodley 2001: 279).

***Odontomyia xanthopus* Bezzi, 1906**

**North African literature records.** Egypt (Woodley 2001: 285; Badrawy 2006: 253).

**World distribution.** Palaearctic: Israel. Afrotropical: Ethiopia, Malawi (Woodley 2001: 285).

####### Genus *OPLODONTHA* Rondani, 1863

***Oplodontha minuta* Fabricius, 1794**

= *Oplodontha oasina* Lindner, 1925

= *Eulalia oasina* Lindner, 1925

**North African literature records.** Egypt: Kharga Oasis, Dakhla Oasis (Lindner 1936: 93–94; Woodley 2001: 287); Egypt (Badrawy 2006: 253).

**World distribution.** Socotra Island (Tkoč and Rozkošný 2014: 431–437).

***Oplodontha viridula* (Fabricius, 1775)**

= *Stratiomys viridula* Fabricius, 1775

= *Stratiomys canina* Panzer, 1798

= *Stratiomys jejuna* Schrank, 1803

= *Muscajejuna Schrank* in Gistl, 1837

= *Odontomyia dentata* Meigen, 1804

= *Odontomyia holosericea* Olivier, 1811

= *Odontomyia lunata* Olivier, 1811

= *Stratiomys subvittata* Meigen, 1822

= *Stratiomys bimaculata* Meigen, 1835

= *Stratiomys bimaculata* Meigen, 1838

= *Odontomyia personata* Loew, 1846

= *Odontomyia interrupta* Loew, 1846

= *Odontomyia heydenii* Jaennicke, 1866

= *Odontomyia atrata* Verrall, 1909

**North African literature record.** Algeria (Woodley 2001: 288).

**World distribution.** Albania, Austria, Belgium, Bulgaria, China, Czech Republic, Denmark, England, France, Finland, Germany, Greece, Hungary, Iraq, Ireland, Israel, Italy, Kazakhstan, Kyrgyzstan, Mongolia, Netherlands, Norway, Poland, Romania, Russia, Slovakia, Spain, Sweden, Switzerland, Turkey (Woodley 2001: 288); Sardinia (Mason et al. 2009: 522).

####### Genus *STRATIOMYS* Geoffroy, 1762

***Stratiomyia africana* Szilady, 1941**

**World distribution.** Only known in North Africa from Algeria: Biskra (Woodley 2001: 296).

***Stratiomys cenisia* Meigen, 1822**

= *Stratiomys flaviventris* Loew, 1846

= *Stratiomyia ahngeri* Pleske, 1901

= *Stratiomyia cypria* Pleske, 1902

= *Stratiomyia kervillei* Villeneuve, 1911

= Stratiomys hispanica ssp. planes James, 1941

**North African literature records.** Morocco: Middle Atlas, Meknès, Rabat, Timahdit, Tangier (Séguy 1930: 64); Algeria: Surcouf (Séguy 1930: 64); Algeria, Egypt, Morocco, Tunisia (Woodley 2001: 297); Egypt (Badrawy 2006: 253).

**World distribution.** Armenia, Austria, Bulgaria, Cyprus, Czech Republic, France, Germany, Hungary, Iran, Israel, Italy, Kazakhstan, Poland, Romania, Russia, Slovakia, Spain, Syria, Turkey, Turkmenistan, Ukraine, Yugoslavia (Woodley 2001: 297).

***Stratiomys deserticolor* Lindner, 1930**

**World distribution.** Only known in North Africa from Egypt: Siwa Oasis (Woodley 2001: 298); (Badrawy 2006: 254).

***Stratiomys hispanica* Pleske, 1901**

**North African literature record.** Algeria (Woodley 2001: 300).

**World distribution.** France, Spain (Woodley 2001: 300).

***Stratiomys longicornis* (Scopoli, 1763)**

= *Hirtea longicornis* Scopoli, 1763

= *Musca tenebricus* M. Harris, 1778

= *Stratiomys strigata* Fabricius, 1781

= *Stratiomys tomentosa* Schrank, 1803

= *Stratiomys villosa* Meigen, 1804

= *Stratiomys nubeculosa* Meigen, 1804

= *Stratiomys thoracica* Fabricius, 1805

= *Stratiomys hirtuosa* Meigen, 1830

= *Stratiomys anubis* (Wiedemann, 1830)

= *Stratiomyia flavifrons* Macquart, 1838

= Stratiomys strigata var. pallida Loew, 1840

= *Stratiomys lambessiana* (Bigot, 1879) (Lindner 1936: 62)

= *Stratiomys flavo limbata* (A. Costa, 1893) (Lindner 1936: 60)

= *Stratiomyia segnis* (Becker, 1906)

= *Hirtea efflatouni* (Lindner, 1925) (Lindner 1936: 58-59)

= Stratiomyia (Hirtea) surcoufi (Séguy, 1930) (Lindner 1936: 64)

= *Hirtea surcoufi* (Séguy, 1932)

= Stratiomyia longicornis ssp. palaestinensis Lindner, 1937

= Stratiomyia (Hirtea) longicornis ssp. flavoscutellata Lindner, 1940

**North African literature records.** Morocco: Casablanca (Séguy 1930: 63); Morocco, Algeria: Lambessa, Tunisia (Lindner 1936: 60, 62, 64); Algeria, Egypt, Morocco, Tunisia (Woodley 2001: 301); Egypt (Badrawy 2006: 254).

**World distribution.** Afghanistan, Albania, Armenia, Austria, Azerbaijan, Belgium, Bulgaria, China, Cyprus, Czech Republic, Denmark, England, France, Germany, Greece, Hungary, Iran, Israel, Italy, Korea, Lithuania, Malta, Mongolia, Netherlands, Poland, Portugal, Romania, Russia, Scotland, Slovakia, Slovenia, Spain, Sweden, Switzerland, Tunisia, Turkey, Yugoslavia (Woodley 2001: 301); Sardinia (Mason et al. 2009: 524).

***Stratiomys singularior* (Harris, 1776)**

= *Musca singularius* Harris, 1776

= *Stratiomys furcata* Fabricius, 1794

= *Stratiomys panthaleon* Fallen, 1817

= *Stratiomys riparia* Meigen, 1822

= *Stratiomys paludosa* Siebke, 1863

**North African literature record.** Egypt (Badrawy 2006: 254).

**World distribution.** Armenia, Austria, Belgium, Bulgaria, China, Czech Republic, Denmark, England, Estonia, Finland, France, Germany, Hungary, Iran, Ireland, Italy, Kazakhstan, Lithuania, Mongolia, Netherlands, Norway, Poland, Romania, Russia, Slovakia, Spain, Sweden, Switzerland, Ukraine, Yugoslavia (Woodley 2001: 301).

##### Subfamily NEMOTELINAE Kertesz, 1912

###### Genus *LASIOPA* Brulle, 1832

***Lasiopa benoisti* Séguy, 1930**

**World distribution.** Only known in North Africa from Morocco: Meknès and Algeria: Boghari (Séguy 1930: 60); Morocco: Meknès, Algeria: Boghari, Bougie (Woodley 2001: 309).

***Lasiopa manni* Mik, 1882**

**North African literature records.** Algeria: Constantine (Becker and Stein 1912: 63); Tunisia (Woodley 2001: 309).

**World distribution.** Italy, Turkey (Woodley 2001: 309).

***Lasiopa pantherina* Séguy, 1930**

**World distribution.** Only known in North African from Morocco: Maharidja (Séguy 1930: 62; Woodley 2001: 309).

###### Genus *NEMOTELUS* Geoffroy, 1762

####### Subgenus Nemotelus Geoffroy, 1762

***Nemotelus anchora* Loew, 1846**

= *Nemotelus siculus* Jaennicke, 1866

= *Nemotelus persicus* Pleske *in* Lindner, 1937

**North African literature records.** Algeria, Tunisia (Woodley 2001: 311); Egypt (Badrawy 2006: 252); Egypt: Fayoum (Mohammad et al. 2009: 99)

**World distribution.** Iran, Israel, Italy, Malta, Russia (Woodley 2001: 311); Sardinia (Mason et al. 2009: 510).

***Nemotelus atriceps* Loew, 1856**

= *Nemotelus longicornis* Lindner, 1937

**Figure 17.**
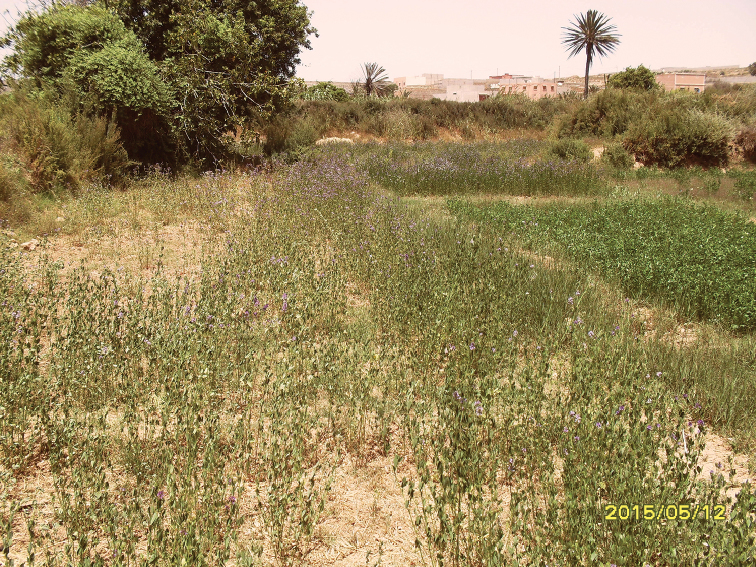
Habitat of *Nemotelus atriceps*: Village Massa.

**New record.** Morocco: Village Massa (Fig. 17), 11.V.2015, 1♂, sweep net, Coll. Yimlahi and Belqat.

**North African literature records.** Algeria, Tunisia (Woodley 2001: 312).

**World distribution.** France, Portugal, Spain (Woodley 2001: 312).

***Nemotelus beckeri* Hauser, 1998**

**World distribution.** Known in North Africaonly from Algeria and Tunisia: Tabaraka, Khathairie (Hauser 1997: 453; Woodley 2001: 313).

***Nemotelus candidus* Becker, 1906**

**World distribution.** Knownin North Africa only from Algeria: Hammam-Salahin (Lindner 1936: 122–123), Biskra, Hammam-Salahin (Woodley 2001: 314) and Egypt (Badrawy 2006: 252); Egypt: Dekhela Mariout, Wadi El Natroun (Mohammad et al. 2009: 99–100).

***Nemotelus carthaginis* Becker, 1906**

**World distribution.** Only known in North Africa from Tunisia (Lindner 1936: 123), Tunisia: Karthago (Woodley 2001: 314).

***Nemotelus cingulatus* Dufour , 1852**

= *Nemotelus lateralis* Dufour, 1852

= *Nemotelus pulcher* Loew, 1871

= *Nemotelus aemulus* Loew, 1871

= *Nemotelus consimilis* (Becker, 1915)

**Figure 18.**
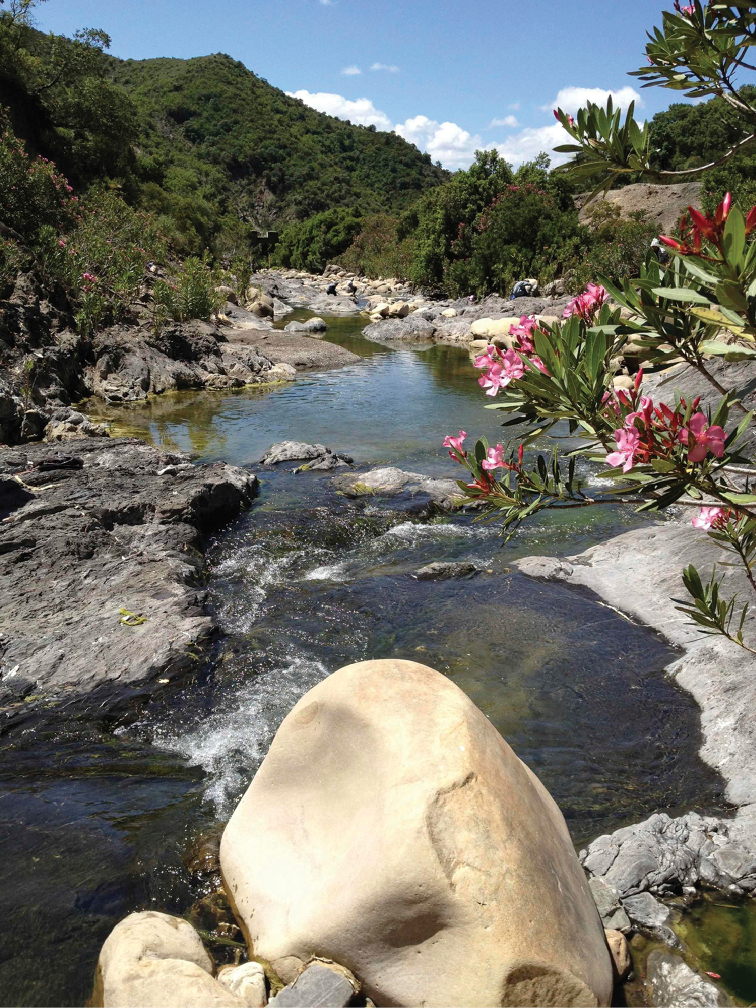
Habitat of *Nemotelus cingulatus*: Oued El Koub environment.

**Figure 19.**
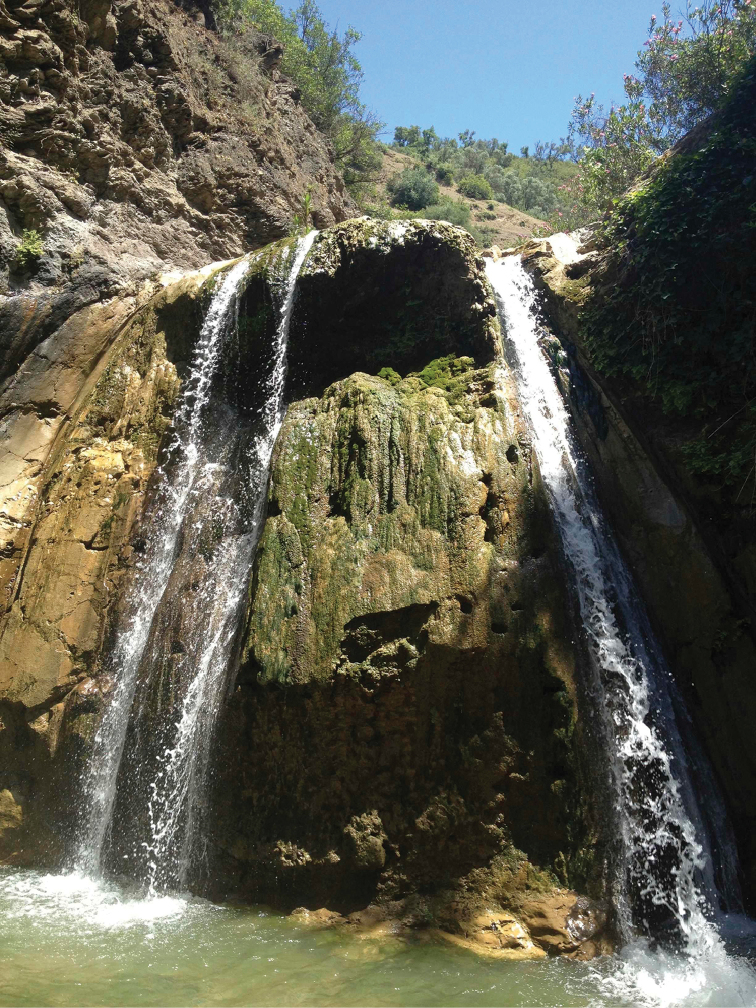
Habitat of *Nemotelus cingulatus*: Oued El Koub.

**New locality.** Morocco, Rif: Daya Afrate (Fig. 15), 2♂♂, 18/IV/2015, sweep net; Oued El Koub (Figs 18, 19), 1♀, 06/V/2015, Coll. Yimlahi and Belqat.

**North African literature records.** Algeria, Tunisia (Lindner 1936: 123–124); Algeria, Morocco, Tunisia (Woodley 2001: 314).

**World distribution.** France, Spain (Woodley 2001: 314).

**Figure 20.**
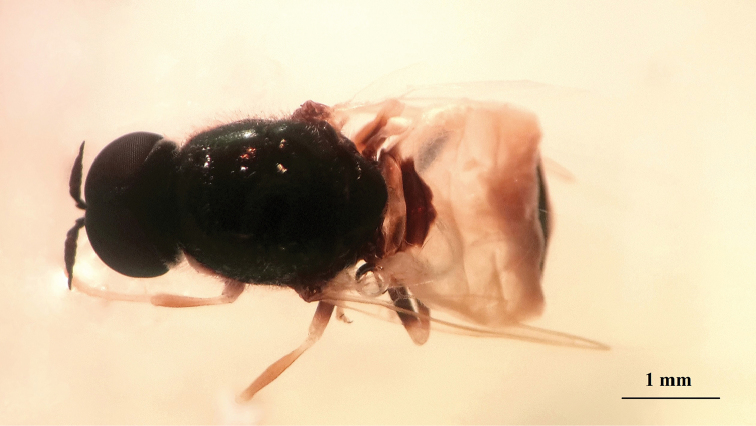
*Nemotelus danielssoni*: Male adult (dorsal view).

**Figure 21.**
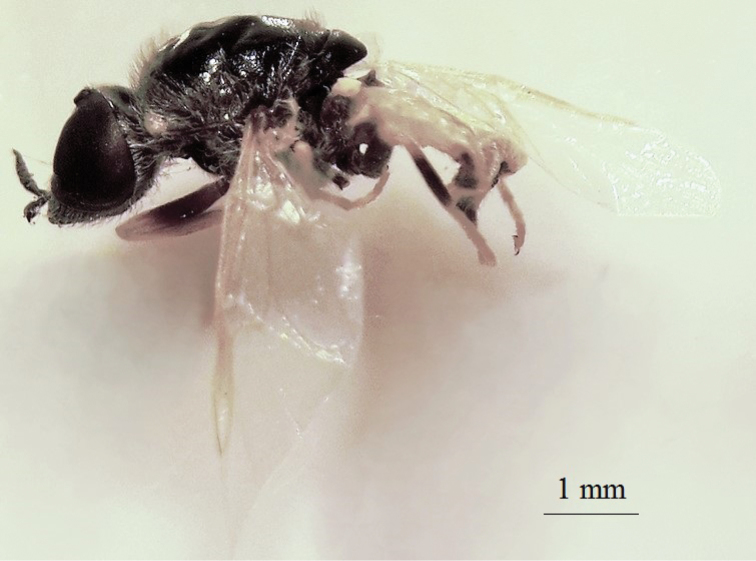
*Nemotelus danielssoni*: Male adult (lateral view).

**Figure 22.**
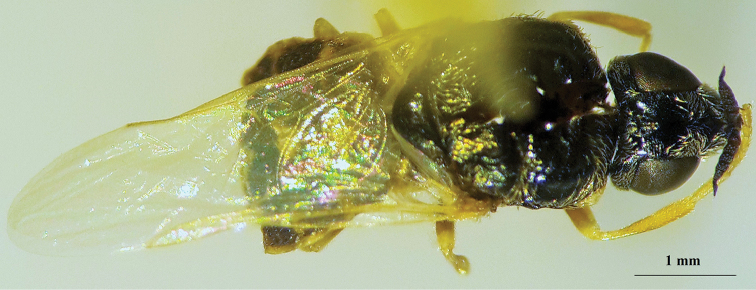
*Nemotelus danielssoni*: Female adult (dorsal view).

**Figure 23.**
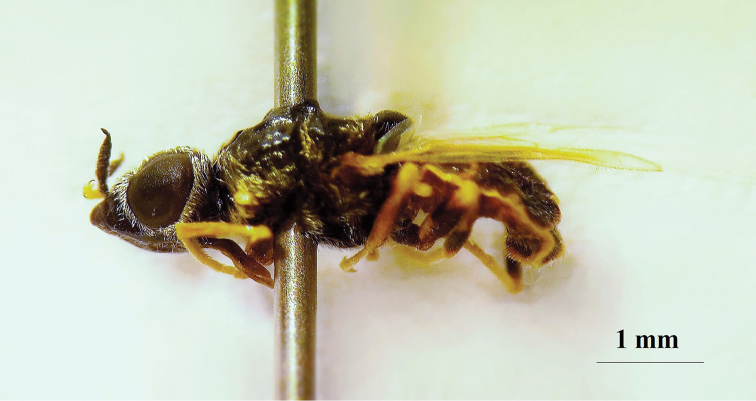
*Nemotelus danielssoni*: Female adult (lateral view).

***Nemotelus danielssoni* Mason, 1989**

(Figs 20, 21, 22, 23)

**World distribution.** Greece (Woodley 2001: 315).

**Figure 24.**
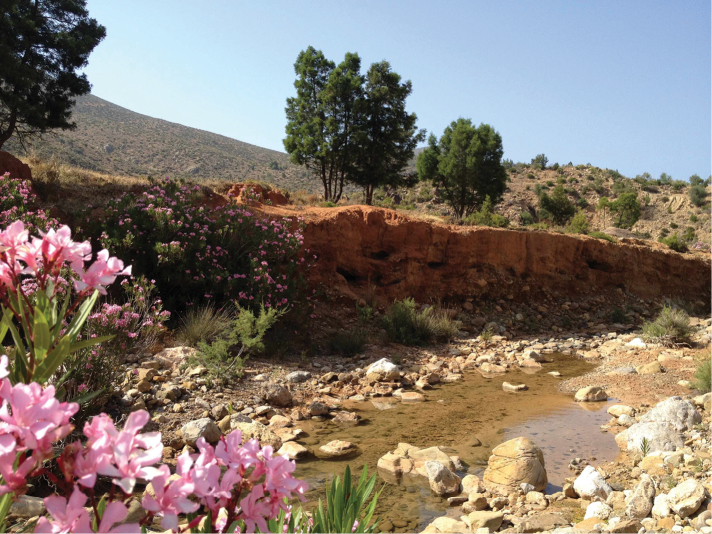
Habitat of *Nemotelus danielssoni*: Oued Izelfane.

**New record.** Morocco: Oued Izelfane (Fig. 24), 8♂♂12♀♀, 25/VI/2013, sweep net, Coll. Yimlahi and Belqat.

*Nemotelus danielssoni* was described as a new species only from the male specimen (from Crete Island, Greece). Recently, Mason and Rozkošný (2003) have described the female.

The species is recorded from Izelfane in Morocco. This finding is very interesting, as it represents the first record from North Africa of a supposed endemic species of Greece.

***Nemotelus dentatus* Becker, 1902**

**World distribution.** Only known in North Africa from Egypt: El Alagto Marg (Lindner 1925), Birket-el-Karun (Woodley 2001: 315); (Badrawy 2006: 252); Coastal strip, Lower Nile, Western desert (Mohammad et al. 2009: 100).

***Nemotelus lasiops* Loew, 1846**

= *Nemotelus striativentris* Lindner, 1937

**North African literature record.** Tunisia (Woodley 2001: 317).

**World distribution.** Italy (Woodley 2001: 317); Sardinia (Mason et al. 2009: 512).

***Nemotelus latiusculus* Loew, 1871**

= *Nemotelus cothurnatus* Bigot, 1879

= *Nemotelus cardinalii* Bezzi, 1898

= *Nemotelus perplexus* Becker, 1915

= *Nemotelus freidbergi* Lindner, 1975

**Figure 25.**
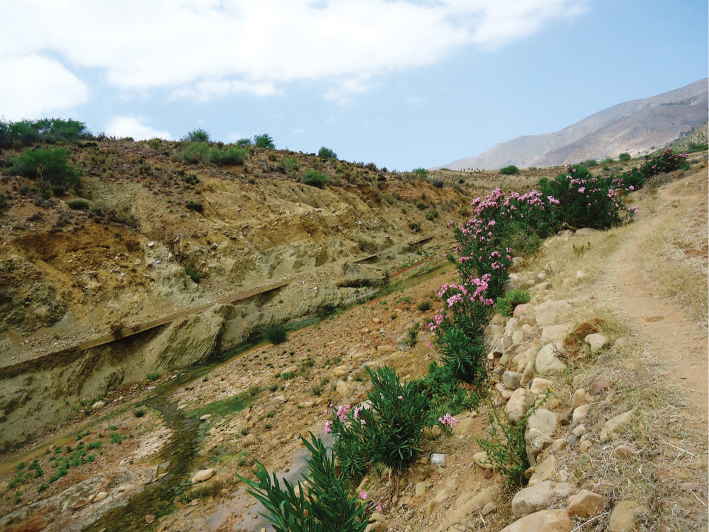
Habitat of *Nemotelus latiusculus*: Barrage Moulay Bouchta.

**New locality.** Morocco, Rif: Barrage Moulay Bouchta (Fig. 25), 1♂, 05/IV/2014, sweep net, Coll. Yimlahi and Belqat.

**North African literature records.** Tunisia (Lindner 1936: 137); Algeria, Morocco, Tunisia (Woodley 2001: 318).

**World distribution.** Israel, Italy, Spain (Woodley 2001: 318).

***Nemotelus longirostris* Wiedemann, 1824**

= *Nemotelus gadensis* Schiner, 1868

= *Nemotelus pilosus* Loew, 1871

= *Nemotelus fuscinervis* Loew, 1871

**North African literature records.** Morocco: Tangier (Becker and Stein 1912: 62; Séguy 1930: 59); Morocco, Algeria (Lindner 1936: 131); Algeria, Morocco, Tunisia (Woodley 2001: 318).

**World distribution.** France, Spain (Woodley 2001: 318).

***Nemotelus maculiventris* Bigot, 1861**

= *Nemotelus andalusiacus* Lindner, 1937

**Figure 26.**
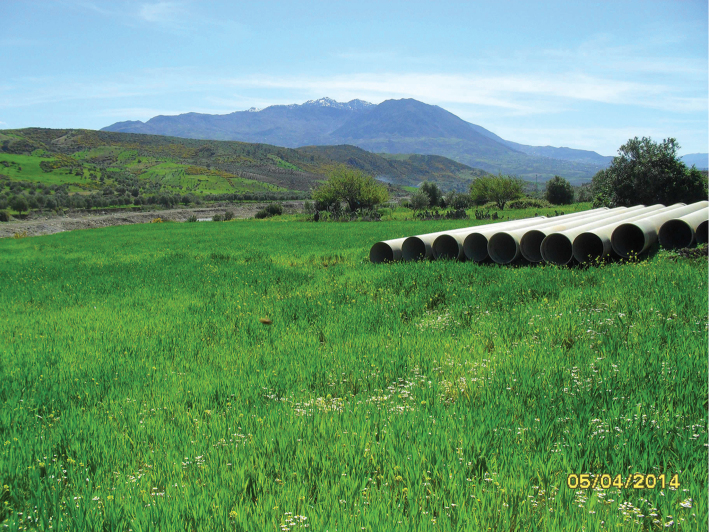
Habitat of *Nemotelus maculiventris* (Bigot, 1861): Oued Zandoula.

**New record.** Morocco, Rif: Oued Zandoula (Fig. 26), 1♂2♀♀, 06/V/2015, sweep net, coll. Yimlahi and Belqat.

**North African literature record.** Algeria (Woodley 2001: 318).

**World distribution.** Italy, Spain (Woodley 2001: 318).

***Nemotelus marinus* Becker, 1902**

**World distribution.** Only known in North Africa from Egypt (Badrawy 2006: 254); Egypt: Suez (Woodley 2001: 318), Fayed, Ferdan, Ismailia, Wadi Hoff, Wadi El Natroun (Mohammad et al. 2009: 100–101).

***Nemotelus matrouhensis* Mohammad, Fadl, Gadalla & Badrawy, 2009**

**World distribution.** Only known in North Africa from Egypt (Mohammad et al. 2009: 100–101).

***Nemotelus nigrifrons* Loew, 1846**

= *Nemotelus tomentosus* Becker, 1906

**Figure 27.**
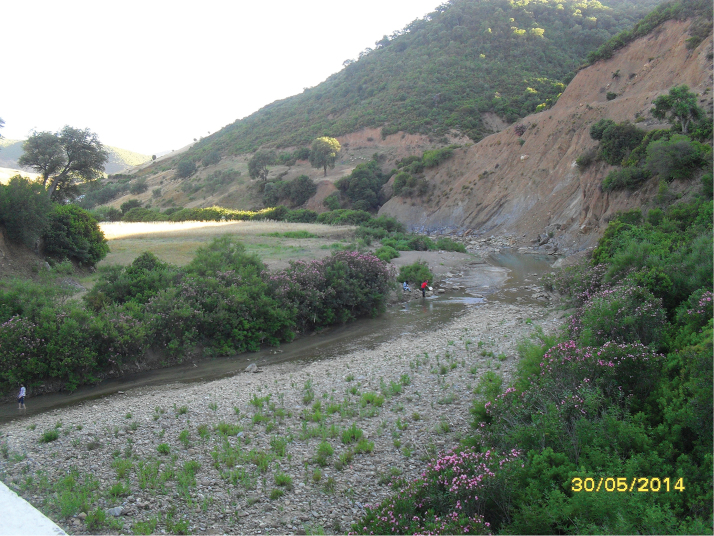
Habitat of *Nemotelus nigrifrons*: Affluent Oued Tarmast.

**New locality.** Affluent Tarmast (Fig. 27), 2♂♂1♀, 26/VI/2013, sweep net, coll. Yimlahi and Belqat.

**North African literature records.** Morocco: Tangier (Becker and Stein 1912: 62); Morocco, Algeria, Tunisia (Lindner 1936); Algeria, Libya, Morocco, Tunisia (Woodley 2001: 319).

**World distribution.** Israel, Italy (Woodley 2001: 319); Sardinia (Mason et al. 2009: 512).

***Nemotelus niloticus* Olivier, 1811**

= *Nemotelus albifacies* Becker, 1902 syn. n.

= *Nemotelus duofasciatus* Woodley 2001

= *Nemotelus fasciatus* Olivier, 1811

= *Nemotelus oasis* Becker, 1906

= *Nemotelus theodori* Lindner, 1974

**North African literature records.** Algeria: Biskra (Lindner 1936: 117); Egypt: Alexandria (Woodley 2001: 311), Egypt (Badrawy 2006: 254), Dekhela Mariout, Fayid, Fayoum, Gabal Asfer, Dekhela, Ismailia, Mallaha Mariout, Ramleh, Sherbin, Zaranik protectorate (Mohammad et al. 2009: 98–101).

**World distribution.** United Arab Emirates (Hauser 2008: 598); Sardinia (Mason et al. 2009: 512).

***Nemotelus notatus* Zetterstedt, 1842**

= *Nemotelus brachystomus* Loew, 1846

= *Nemotelus nigroaeneus* Verhoeff, 1891

= *Nemotelus punctiventris* Becker, 1902

= Nemotelus brachystomus form aegyptiacus (Lindner, 1925)

= *Nemotelus balearicus* Lindner, 1937

= *Nemotelus zernyi* Lindner, 1937

**North African literature record.** Egypt (Badrawy 2006: 254), Egypt: Coastal strip (Mohammad et al. 2009: 102–103).

**World distribution.** Austria, Belgium, Denmark, England, Finland, France, Germany, Ireland, Netherlands, Norway, Poland, Spain, Sweden (Woodley 2001: 313); Sardinia (Mason et al. 2009: 515–520); Turkey (Üstüner et al. 2002: 21).

***Nemotelus pantherinus* (Linnaeus, 1758)**

= *Musca pantherina* Linnaeus, 1758

= *Stratiomys albipes* Geoffroy in Fourcroy, 1785

= *Stratiomys marginellus* Thunberg, 1789

= *Musca marginella* Gmelin, 1790

= *Nemotelus nigritus* Meigen, 1804

= *Nemotelus marginellus* Fallen, 1817

= *Nemotelus nigritus* Meigen, 1822

= *Nemotelus Jraternus* Loew, 18 46

= *Nemotelus gracilis* Loew, 1846

= *Nemotelus satunini* Pleske in Lindner, 1937

= *Nemotelus zelleri* Pleske in Lindner, 1937

= *Nemotelus albirostris* Szilady, 1941

= *Nemotelus caucasicus* Nartshuk, 1969

= *Nemotelus punctirostris* Lindner, 1974

**North African literature record.** Morocco: Tangier (Séguy 1930: 59), Morocco (Woodley 2001: 320).

**World distribution.** Albania, Armenia, Austria, Azerbaijan, Belgium, Bulgaria, Cyprus, Czech Republic, Denmark, England, Estonia, France, Germany, Greece, Hungary, Ireland, Israel, Italy, Latvia, Netherlands, Norway, Poland, Romania, Russia, Slovakia, Spain, Sweden, Switzerland, Tajikistan, Turkey, Yugoslavia (Woodley 2001: 320); Sardinia (Mason et al. 2009: 521).

***Nemotelus proboscideus* Loew, 1846**

= *Nemotelus punctatus* Fabricius, 1794

= *Nemotelus algericus* Jaennicke, 1866

**North African literature records.** Morocco, Tunisia (Lindner 1936: 139); Algeria, Libya, Tunisia, omitted in Morocco by Woodley (2001: 321).

**World distribution.** Italy (Woodley 2001: 321).

***Nemotelus punctiventris* Becker 1902**

**World distribution.** Only known in North Africa from Egypt (Mohammad et al. 2009: 100–101).

***Nemotelus subuliginosus* Rozkosny, 1974**

**World distribution.** Only known in North Africa from Morocco: Tangier (Woodley 2001: 322).

***Nemotelus ventralis* Meigen, 1830**

**World distribution.** Only known in North Africa from Morocco (Lindner 1936: 146), Morocco: Essaouira (Woodley 2001: 323).

####### Subgenus Camptopelta (Williston, 1917)

***Nemotelus nigrinus* Fallen, 1817**

= *Nemotelus carneus* Walker, 1849

= *Nemotelus crassus* Loew, 1863

= *Nemotelus unicolor* Loew, 1863

= *Nemotelus carbonarius* Loew, 1869

**North African literature record.** Morocco (Woodley 2001: 326).

**World distribution.** Nearctic: Canada, USA. Neotropical: Mexico. Palaearctic: Afghanistan, Austria, Azerbaijan, Belgium, Bulgaria, China, Czech Republic, Denmark, England, Estonia, Finland, Germany, Hungary, Ireland, Latvia, Lithuania, Mongolia, Netherlands, Norway, Poland, Romania, Russia, Slovakia, Spain, Sweden, Switzerland, Tibet, Ukraine, Yugoslavia (Woodley 2001: 326), Turkey (Üstüner 2010: 110).
